# Vital signs assessed in initial clinical encounters predict COVID-19 mortality in an NYC hospital system

**DOI:** 10.1038/s41598-020-78392-1

**Published:** 2020-12-09

**Authors:** Elza Rechtman, Paul Curtin, Esmeralda Navarro, Sharon Nirenberg, Megan K. Horton

**Affiliations:** 1 Department of Environmental Medicine and Public Health, Icahn School of Medicine at Mount Sinai, One Gustave Levy Place, Box 1057, New York, NY 10029 USA; 2 Scientific Computing, Icahn School of Medicine at Mount Sinai, New York, NY USA

**Keywords:** Diseases, Medical research, Public health

## Abstract

Timely and effective clinical decision-making for COVID-19 requires rapid identification of risk factors for disease outcomes. Our objective was to identify characteristics available immediately upon first clinical evaluation related COVID-19 mortality. We conducted a retrospective study of 8770 laboratory-confirmed cases of SARS-CoV-2 from a network of 53 facilities in New-York City. We analysed 3 classes of variables; demographic, clinical, and comorbid factors, in a two-tiered analysis that included traditional regression strategies and machine learning. COVID-19 mortality was 12.7%. Logistic regression identified older age (OR, 1.69 [95% CI 1.66–1.92]), male sex (OR, 1.57 [95% CI 1.30–1.90]), higher BMI (OR, 1.03 [95% CI 1.102–1.05]), higher heart rate (OR, 1.01 [95% CI 1.00–1.01]), higher respiratory rate (OR, 1.05 [95% CI 1.03–1.07]), lower oxygen saturation (OR, 0.94 [95% CI 0.93–0.96]), and chronic kidney disease (OR, 1.53 [95% CI 1.20–1.95]) were associated with COVID-19 mortality. Using gradient-boosting machine learning, these factors predicted COVID-19 related mortality (AUC = 0.86) following cross-validation in a training set. Immediate, objective and culturally generalizable measures accessible upon clinical presentation are effective predictors of COVID-19 outcome. These findings may inform rapid response strategies to optimize health care delivery in parts of the world who have not yet confronted this epidemic, as well as in those forecasting a possible second outbreak.

## Introduction

Identifying susceptibility to COVID-19 related mortality based on measures immediately available at the first clinical evaluation may assist medical staff in providing timely and effective care for each patient. To date, many of the descriptions of COVID-19 patients rely on small datasets which may suffer from overfitting, limiting the generalization of the findings to other populations^1–6^. Further, most existing studies of COVID-19 related health outcomes compile demographic statistics offering information only about single risk factors, rather than combined risk^7–9^. Very few studies^10,11^ have taken a comprehensive risk evaluation based on personalized demographic and physical characteristics acquired at the first encounter to predict COVID-19 related mortality.

In the present study, we explicitly test how demographic, clinical, and co-morbid disease factors relate to COVID-19 mortality in 8770 patients with laboratory-confirmed SARS-CoV-2 infection. We analysed 3 broad classes of variables; demographic factors, clinical indicators, and comorbid conditions, in a two-tiered analysis that included traditional regression strategies and machine learning methodologies. To provide timely information that would support fast clinical decision-making, we focused on factors that can be assessed immediately at the first clinical evaluation and did not require laboratory processing or extensive medical chart review.

## Methods

### Data collection

We conducted a retrospective observational study using a de-identified data set of all COVID-19 related encounters at all Mount Sinai Health System facilities (n = 53) in New York City. As of April 24, 2020, nearly 47,000 patients were tested for COVID-19 or under investigation for COVID-19. For this analysis, we included all cases (n = 8770) confirmed SARS-CoV-2 positive by real time-polymerase chain reaction (RT-PCR) in nasopharyngeal or oropharyngeal swabs collected in outpatient, urgent care, emergency, and inpatient facilities. Demographic and clinical data were extracted from Epic electronic health record (Verona, WI) databases and deidentified^12^.

Our dataset included: (1) demographic variables [age, sex, race, ethnicity and smoking status and body mass index (BMI)]; (2) clinical variables [heart rate, temperature, respiratory rate, oxygen (O2) saturation], and (3) comorbid conditions [chronic kidney disease (CKD), asthma, chronic obstructive pulmonary disease (COPD), hypertension, diabetes, human immunodeficiency virus (HIV) and cancer]. Temperature, O_2_ saturation, heart rate and respiratory rate refer to initial vital signs taken during the patient encounter. This study was approved by the Institutional Review Board of the Icahn School of Medicine at Mount Sinai. The dataset had no identifiers and was considered Non-Human Subject Research (NHSR) and thus the need for patient consent was waived.

### Descriptive and inferential modelling

Sample size was determined by the number of SARS-CoV-2 positive patients treated by Mount Sinai Health System during the study period (Date to April 24, 2020) and we did not perform a priori statistical sample size calculation. A multivariable logistic regression model with the binary outcome (survivor/non-survivor) was used to estimate the association between COVID-19 related mortality and baseline demographic, clinical characteristics, and comorbidities. Age was modelled as a decadal continuous variable to increase the interpretability of the results. Race and ethnicity were collected as separate variables and combined into 4 categories: Black, Hispanic, White, and other/Unknown, where patients with Hispanic ethnicity were grouped in the race category 'Hispanic', regardless of their race classification. Patients with oxygen saturation inferior to 40% were excluded, to eliminate this variable as the cause of death. Smoking was collapsed into two categories: ever/never. Odds ratios (OR) for mortality relative to each predictor were estimated, and statistical significance was assessed relative to an alpha of 0.05. Models were implemented in R (v3.5.1) using the *glm* package.

### Predictive modelling

To assess the utility of these measures in predicting COVID-19 mortality, we constructed a machine learning model utilizing the Extreme Gradient Boosting framework implemented in the *Xgboost* (v1.0.0.2) package in R. The available data were divided in a training set (60% of data) and a holdout test set. In the training set, the *mlr* (v2.17.1) package was used to tune model hyperparameters across tenfold stratified cross-validation with a random grid search. Hyperparameters used for tuning included the boosting function (linear, or tree-based), tree depth, learning rate, L1 regularization parameter, L2 regularization parameter, boosting rounds, class weighting, and the minimum loss reduction for partitioning. Following training, overall model performance was evaluated by predicting mortality in the naïve holdout test set, with receiver operating characteristic (ROC) curves and area-under-curve (AUC) used to assess model efficacy. Feature importance was estimated through the calculation of gain, which reflects the fractional contribution of a given feature to the overall model.

## Results

As of April 24, 2020, a total of 46,945 patients had an encounter at a Mount Sinai facility who have either been tested for COVID-19 or who are under investigation for COVID-19. RT-PCR confirmation for SARS-CoV-2 was available for 8770 of these patients which comprise the final sample for our analyses. Overall, 4766 (54.3%) of patients were male, 4525 (70.1%) never smoked, 3996 (69.2%) had a BMI greater than 25. Self-reported race/ethnicity included 2310 (26.4%) White, 1955 (22.3%) Black, and 1975 (22.5%) Hispanic. The median age was 60 years (IQR, 32–88) (range, 0–90 years). A total of 2293 (26.1%) were aged 71 years and older, and 2956 (33.7%) were younger than 51 years. The most common comorbidities were hypertension (2281, 26%), and diabetes (1631, 18.6%). At encounter, 784 (11.5%) presented with a respiratory rate greater than 24 breaths/min, 1308 (18.4%) with temperature greater than 38.0 °C, 2582 (36.6%) with heart rate greater than 100 beats/min, and 2826 (40.4%) with oxygen saturation level below 96%. Among the confirmed cases included in our analyses, 1114 (12.7%) died from COVID-related symptoms. For non-survivors, the median time of death after the encounter was 6 days. For survivors, the median time of discharge after the encounter was 3 days. Sociodemographic, clinical characteristics, and comorbidities of patients stratified by survival are reported in Table 1.

**Table 1:** Baseline characteristics of 8770 confirmed COVID-19 patients stratified by survival.

Characteristic	All patients (n = 8770)	Survivors (n = 7656)	Non-survivors (n = 1114)
**Demographics**^**a**^			
Sex; n (%)			
Female	4004 (45.7)	3560 (46)	444 (40)
Male	4766 (54.3)	4096 (54)	670 (60)
Age (years); Median (IQR) [range]	60 (44–72) [0–90]	57 (41–69) [0–90]	76 (65–85) [29–90]
Smoking; n (%)			
Never	4525 (71)	3991 (71.9)	534 (64.3)
Yes/former	1853(29)	1557 (28.1)	296 (35.7)
Race/ethnicity; n (%)			
White	2310 (26.4)	1984 (25.9)	326 (29.3)
Black	1955 (22.3)	1693 (22.1)	262 (23.5)
Hispanic	1975 (22.5)	1744 (22.8)	231 (20.7)
Other/unknown	2527 (28.8)	2232 (29.2)	295 (26.5)
BMI; mean (SD) [range]	29 (26–30) [15–83]	28 (24–32) [15–83]	28 (24–33) [16–70]
**Clinical factors**^**b**^			
Heart rate; Mean (SD) [Range]	94 (82–107) [15–206]	94 (82–107) [15–206]	96 (82–111) [28–177]
Temperature; Mean (SD) [range]	37 (37–38) [31–41]	37 (37–38) [31–41]	37 (37–38) [32–41]
Respiratory rate; Mean (SD) [range]	19 (18–20) [10–107]	18 (18–20) [10–107]	20 (18–25) [12–60]
0_2_ Saturation; Mean (SD) [range]	96 (94–98) [40–100]	97 (94–99) [42–100]	94 (89–97) [40–100]
**Comorbidities**^**c**^			
Hypertension; n (%)	2281 (26)	1827 (23.9)	454 (40.7)
CKD; n (%)	753 (8.6)	576 (7.5)	177 (15.9)
Diabetes; n (%)	1631 (18.6)	1325 (17.3)	306( 27.5)
COPD; n (%)	222(2.5)	160 (2.1)	62 (5.6)
HIV; n (%)	139 (1.6)	123 (1.6)	16 (1.4)
Cancer; n (%)	649 (7.4)	561 (7.3)	88 (7.9)
Obesity; n (%)	616 (7)	530 (6.9)	86 (7.7)
Asthma; n (%)	394 (4.4)	341 (4.6)	43 (3.9)

*BMI* body mass index, *CKD* chronic kidney disease, *COPD* chronic obstructive pulmonary disease, *HIV* human immunodeficiency virus.

^a^Self reported.

^b^Clinical vitals measured at first encounter.

^c^Assessed based on medical history by International Statistical Classification of Diseases and Related Health Problems, 10th revision (ICD-10) coding.

**Figure 1:**
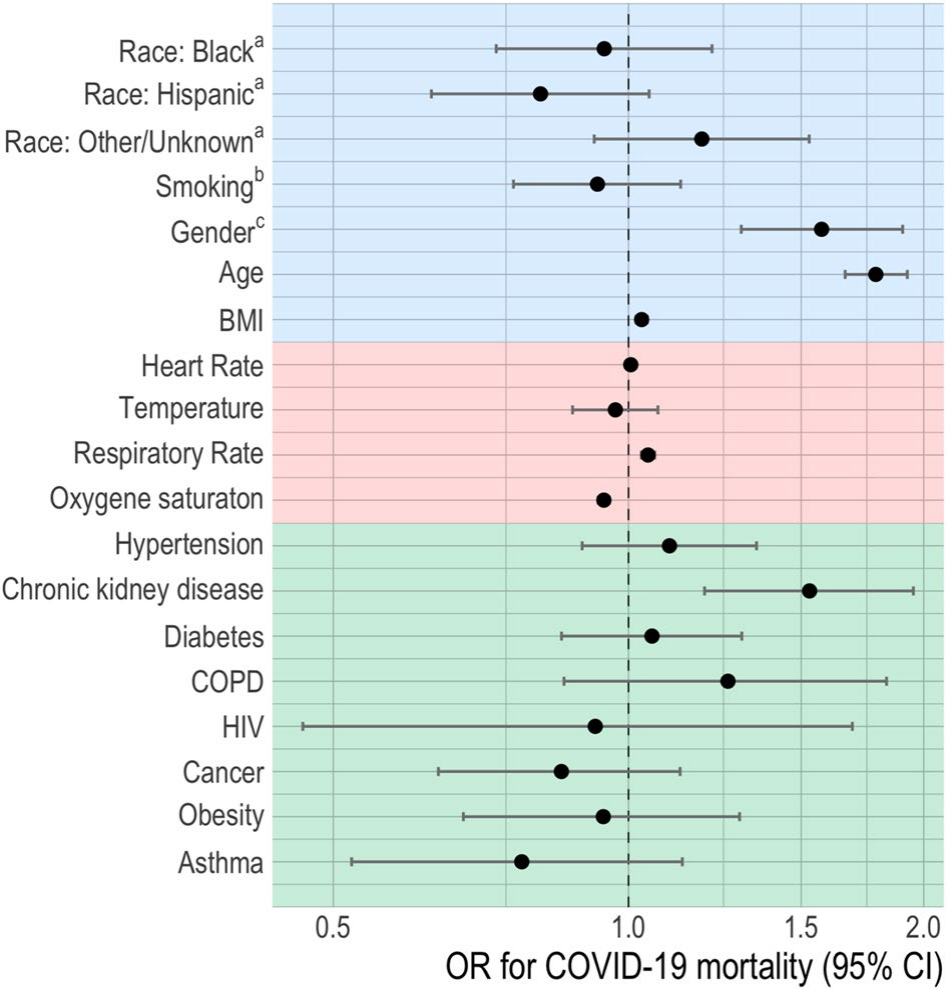
Associations (odds ratio and 95% intervals) of demographics, clinical factors, and comorbidities and COVID-19 mortality. *OR* odds ratio, *BMI *body mass index, *COPD *chronic obstructive pulmonary disease, *HIV *human immunodeficiency virus. Clinical factors were assessed at triage. ^a^In reference to White. ^b^Ever smoked in reference to never smoked. ^c^Males in reference to females. Factors are color coded to indicate the group of characteristics (Blue = Demographics, Red = clinical factors, Green = comorbidities).

Results of the multivariable logistic regression are presented in Fig. 1. Among the demographic variables, age, gender, and BMI were detected as risk factors for COVID-19 mortality; the odds of death increase by 79% per decade of age (OR, 1.79 [95% CI 1.67–1.93]), males had a 58% increase in the odds of death compared to females (OR, 1.58 [95% CI 1.31–1.91]), and the odds of death increase by 3% per increase in BMI point (OR, 1.03 [95% CI 1.02–1.04]). Three baseline clinical characteristics were detected as risk factors for COVID-19 mortality: heart rate (OR, 1.001 [95% CI 1.00–1.01]), respiratory rate (OR, 1.05 [95% CI 1.03–1.06]), and oxygen (O_2_) saturation (OR, 0.94 [95% CI 0.93–0.96]). Among the comorbid conditions, only chronic kidney disease was associated with COVID-19 mortality, increasing the odds of death by 51% (OR, 1.51 [95% CI 1.18–1.93]).

**Figure 2:**
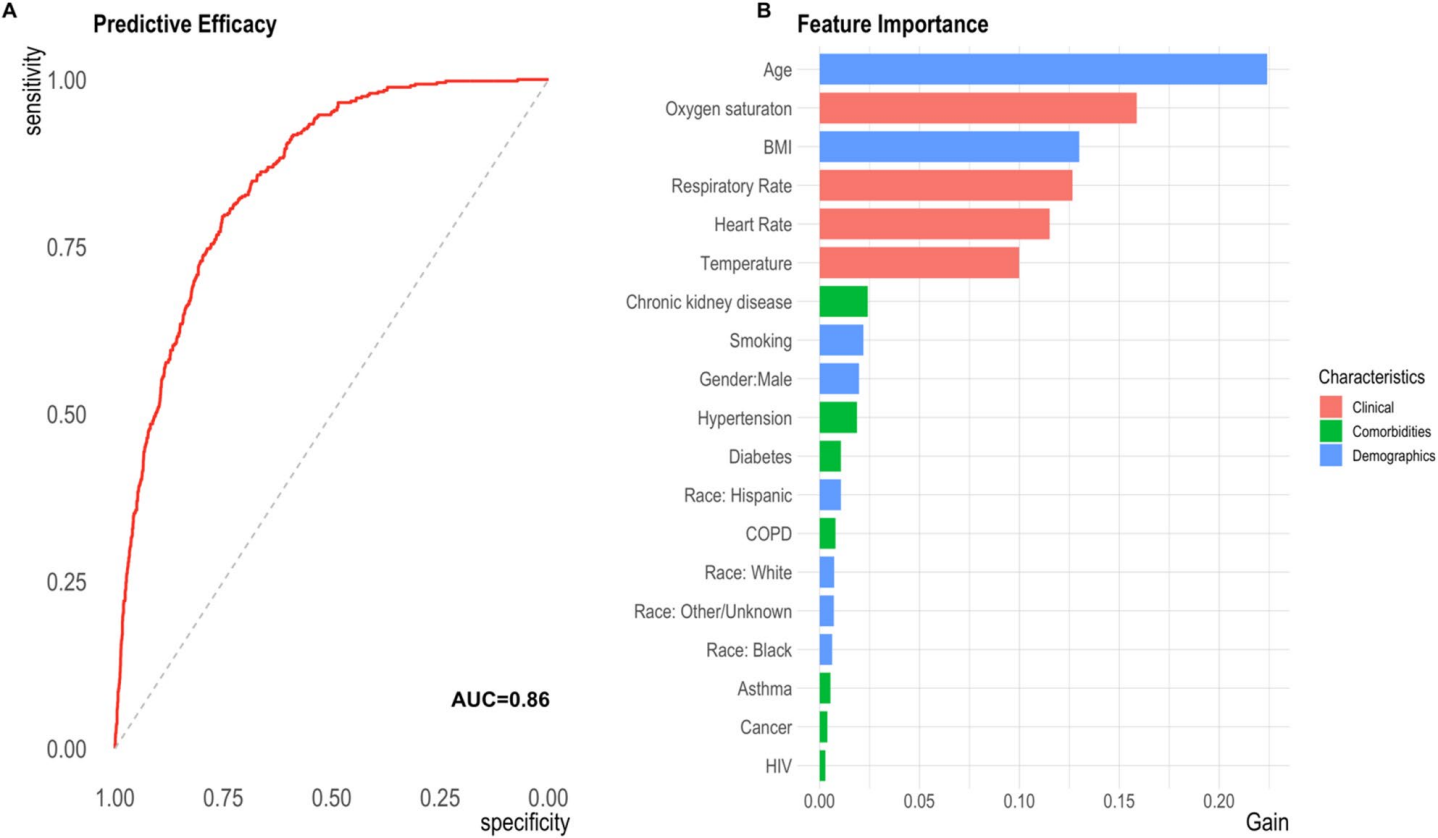
Predictive classification of COVID-19 mortality. **A** Receiver operating characteristic (ROC) curve illustrating the sensitivity and specificity for predicting COVID-19 mortality using a gradient boosting algorithm. **B** Importance of each feature to COVID-19 mortality prediction. Features are color coded to indicate the group of characteristics. *BMI *body mass index, *COPD *chronic obstructive pulmonary disease, *HIV *human immunodeficiency virus. Clinical factors were assessed at the first encounter.

Following cross-validation in a training set, we applied a machine learning model utilizing the extreme gradient boosting framework to a holdout test set. Figure 2, Panel A, shows the receiver operating characteristic (ROC) curve summarizing model performance with an AUC of 0.86. In Fig. 2, Panel B, we show the features that contributed most to model performance, with age, oxygen saturation, BMI, respiratory rate, heart rate, and temperature, contributing most to model importance.

## Discussion

In this study of COVID-19 patients hospitalized in a large, socio-demographically diverse New York City hospital system, we report that vital indicators typically collected during initial clinical evaluations are effective predictors of COVID-19 related mortality. We implemented two approaches to investigate factors of COVID-19 mortality: multivariable logistic regression to describe characteristics associated with mortality, and gradient boosting to predict mortality. Seven clinical factors were associated with COVID-19 mortality: age (older), gender (male), BMI (higher), heart rate (higher), respiratory rate (higher), O_2_ saturation (lower), and chronic kidney disease. When combined, these factors predicted COVID-19 related mortality with an AUC of 0.86 in naïve data following cross-validation in a training set. Age, oxygen saturation, BMI, respiratory rate, heart rate, and temperature contributed most to the prediction of COVID-19 mortality.

This study reports significant associations between vitals measured at the first clinical encounter and COVID-19 mortality. Consistent with earlier reports from China, Italy, and another NYC area, our logistic regression models showed that age, sex, and BMI have a major effect on COVID-19 mortality^7,8,13–15^. Although we confirm previous findings of a high prevalence of hypertension and diabetes in COVID-19 patients^8,13,16^, we did not find significant association between these comorbidities and COVID-19 mortality.

Notably, our results show that immediate, objective measures collected at the time of first clinical presentation can be effective predictors of mortality. Moreover, these measures can be obtained if the patient is unresponsive or unconscious. Our results expand prior descriptive reports to provide statistical confirmation of suspected risk factors and emphasize that the interaction of these variables is ultimately predictive of mortality. These findings may inform rapid response strategies to optimize health care delivery in parts of the world who have yet confronted this epidemic, as well as in those forecasting a possible second outbreak.

This study has several limitations. First, due to a lack of widespread testing for COVID-19, only severe cases of COVID-19 had laboratory confirmation of SARS-CoV-2. As such, this study may have disproportionately included patients with poor outcomes, limiting the generalizability of our study. Second, due to the critical nature of the situation in the New York City area, we did not obtain information regarding oxygen support or ICU admissions. As well, by determining the outcome at the time of analyses, we may have misclassified patients that have not completed their hospital admission. Lastly, given that our analysis focuses specifically on patient characteristics in healthcare facilities, our results should not be interpreted as indicative of patterns in the population at large.

## Conclusions

In this retrospective observational study focusing on demographic and clinical characteristics of confirmed COVID-19 patients in a large NYC hospital system, older age, being a male, higher BMI, presenting vitals of higher heart rate, higher respiratory rate and lower O_2_ saturation as well as having CKD, were identified as risk factors for COVID-19 mortality. We found that these factors could be combined in a gradient-boosting machine learning model to create an effective predictor of mortality with an AUC of 0.86. Notably, our results show that immediate, objective measures collected at the time of clinical presentation, independently of patient level of consciousness, can be effective predictors of mortality. Reliance on results from hematologic and biochemical laboratory tests or extensive medical history review may create a critical lag in response time. These findings may inform rapid response strategies to optimize health care delivery in parts of the world who have yet confronted this epidemic, as well as in those forecasting a possible second outbreak.

## Data Availability

The datasets generated during and/or analysed during the current study are available from the corresponding author on reasonable request.
